# Transcranial Direct Current Stimulation to Enhance Training Effectiveness in Chronic Post-Stroke Aphasia: A Randomized Controlled Trial Protocol

**DOI:** 10.3389/fneur.2019.01089

**Published:** 2019-10-22

**Authors:** Benjamin Stahl, Robert Darkow, Viola von Podewils, Marcus Meinzer, Ulrike Grittner, Thomas Reinhold, Tanja Grewe, Caterina Breitenstein, Agnes Flöel

**Affiliations:** ^1^Department of Neurology, University Medicine Greifswald, Greifswald, Germany; ^2^Department of Neurology, Charité Universitätsmedizin Berlin, Berlin, Germany; ^3^Max Planck Institute for Human Cognitive and Brain Sciences, Leipzig, Germany; ^4^Psychologische Hochschule Berlin, Berlin, Germany; ^5^Institute of Logopedics, FH Johanneum University of Applied Sciences, Graz, Austria; ^6^Centre for Clinical Research, The University of Queensland, Brisbane, QLD, Australia; ^7^Institute of Biometry and Clinical Epidemiology, Charité Universitätsmedizin Berlin, Berlin, Germany; ^8^Berlin Institute of Health, Berlin, Germany; ^9^Institute of Social Medicine, Epidemiology and Health Economics, Charité Universitätsmedizin Berlin, Berlin, Germany; ^10^Faculty of Health and Social Sciences, Fresenius University of Applied Sciences, Idstein, Germany; ^11^Department of Neurology with Institute of Translational Neurology, University of Münster, Münster, Germany; ^12^German Center for Neurodegenerative Diseases, Rostock/Greifswald, Germany

**Keywords:** chronic post-stroke aphasia, intensive speech-language therapy, transcranial direct current stimulation, rehabilitation, randomized controlled trial

## Abstract

**Background:** Intensive speech-language therapy (SLT) can promote recovery from chronic post-stroke aphasia, a major consequence of stroke. However, effect sizes of intensive SLT are moderate, potentially reflecting a physiological limit of training-induced progress. Transcranial direct current stimulation (tDCS) is an easy-to-use, well-tolerated and low-cost approach that may enhance effectiveness of intensive SLT. In a recent phase-II randomized controlled trial, 26 individuals with chronic post-stroke aphasia received intensive SLT combined with anodal-tDCS of the left primary motor cortex (M1), resulting in improved naming and proxy-rated communication ability, with medium-to-large effect sizes.

**Aims:** The proposed protocol seeks to establish the incremental benefit from anodal-tDCS of M1 in a phase-III randomized controlled trial with adequate power, ecologically valid outcomes, and evidence-based SLT.

**Methods:** The planned study is a prospective randomized placebo-controlled (using sham-tDCS), parallel-group, double-blind, multi-center, phase-III superiority trial. A sample of 130 individuals with aphasia at least 6 months post-stroke will be recruited in more than 18 in- and outpatient rehabilitation centers.

**Outcomes:** The *primary* outcome focuses on communication ability in chronic post-stroke aphasia, as revealed by changes on the Amsterdam-Nijmegen Everyday Language Test (A-scale; primary endpoint: 6-month follow-up; secondary endpoints: immediately after treatment, and 12-month follow-up). *Secondary* outcomes include measures assessing linguistic-executive skills, attention, memory, emotional well-being, quality of life, health economic costs, and adverse events (endpoints: 6-month follow-up, immediately after treatment, and 12-month follow-up).

**Discussion:** Positive results will increase the quality of life for persons with aphasia and their families while reducing societal costs. After trial completion, a workshop with relevant stakeholders will ensure transfer into best-practice guidelines and successful integration within clinical routine.

**Clinical Trial Registration:** www.ClinicalTrials.gov, identifier: NCT03930121.

## Introduction

Cerebrovascular diseases are among the most common causes of disability worldwide, and about one third of stroke survivors initially suffer from communication disorders, including aphasia (1). In up to 40% of these individuals, symptoms of aphasia persist 6 months after stroke and rarely recover spontaneously in the ensuing time (2). Critically, chronic post-stroke aphasia affects vocational reintegration, social life, and emotional well-being while placing major burdens on the healthcare system (3).

Meta-analyses concluded that intensive speech-language therapy (SLT) can be effective even in the chronic stage of symptoms (4). In a multi-center randomized controlled trial (RCT), persons with chronic post-stroke aphasia were randomly assigned to either 3 weeks of intensive SLT or a waiting period prior to treatment (5). Verbal communication improved after intensive SLT, but not after the waiting period, as assessed by the Amsterdam-Nijmegen Everyday Language Test (ANELT) (6). The study revealed an average increase of three points on the ANELT (A-scale) immediately after intensive SLT, a relative difference amounting to ~10% (between-group effect: Cohen's *d* = 0.58).

Motivated by a lack of data identified in systematic reviews (7), several proof-of-concept studies provide evidence suggesting that transcranial direct current stimulation (tDCS) is an easy-to-use, well-tolerated, and low-cost approach to boost the effectiveness of SLT in chronic post-stroke aphasia (8). Most of these studies are small-to-medium phase-II trials, with naming ability (9) or other linguistic skills as primary outcomes, but no parameters reflecting everyday life (7), or with individually determined stimulation not feasible for standard application (9–11). Moreover, most of the studies did not assess training effects over extended periods of time.

Our group recently published the first prospective randomized placebo-controlled (using sham-tDCS), parallel-group, double-blind, single-center, phase-II superiority trial on intensive naming therapy combined with anodal-tDCS of the left primary motor cortex (M1), including a sample of 26 persons with post-stroke aphasia, a 6-month follow-up, and outcomes relevant to everyday life (12). Results indicated significantly improved naming and proxy-rated communication ability in both groups. However, treatment gains for trained items were significantly better maintained in the anodal-tDCS group at the 6-months follow-up (Cohen's *d* = 1.19). Importantly, progress in communication ability was significantly higher with anodal-tDCS than with sham-tDCS at all endpoints (Cohen's *d* = 0.75–0.99).

In summary, preliminary data suggest that anodal-tDCS can benefit naming and communication ability in chronic post-stroke aphasia, with medium-to-large effect sizes and long-term stability of treatment gains. To ensure transfer of the intervention to best-practice guidelines and successful integration within clinical routine, a phase-III RCT is needed with adequate power, ecologically valid outcomes, and evidence-based SLT (5).

## Materials and Methods

### Design

The planned study DC-TRAIN-APHASIA (transcranial direct current stimulation to enhance training effectiveness in chronic post-stroke aphasia) is a prospective randomized placebo-controlled (using sham-tDCS), parallel-group, double-blind, multi-center, phase-III superiority trial.

### Patient Involvement and Ethics Approval

Patients, relatives, and their representatives were given the opportunity to discuss the study protocol and utter concerns not addressed in a draft proposed at the time. It was pointed out that in- and exclusion criteria should reflect realistic routine-healthcare conditions; we followed this advice, whenever possible. The final protocol was approved by the Ethics Review Board of the University Medicine Greifswald, Germany (reference number: BB-013/18). Written informed consent will be obtained from each participant. All study procedures are in accordance with the Declaration of Helsinki in its current version (for details, see www.wma.net).

### Recruitment and Setting

Individuals with chronic post-stroke aphasia will be recruited in more than 18 in- and outpatient rehabilitation centers throughout Germany (for an updated list of study sites, see record on ClinicalTrials.gov; identifier: NCT03930121). A similar network of rehabilitation centers proved to be time- and cost-effective in a previous large-scale RCT with long-term follow-ups (5).

### In- and Exclusion Criteria

*Inclusion criteria* are: Left-hemisphere cortical or subcortical stroke with first-ever aphasic symptoms; at least 6 months post-onset of stroke; fluent or non-fluent aphasia, as determined by the Aachen Aphasia Test (AAT) (13); moderate-to-severe word finding difficulties (maximum of 60% correct items on a computerized naming task before treatment); at least one correct reaction on the first part of the AAT subscale Token Test (ensuring basic comprehension skills); at least one point on the communicative task of the AAT subscale Spontaneous Speech (ensuring basic communication abilities); German as first language; age range 18–70 years; and intact left-hemisphere “hand knob” and underlying white matter (for placement of anode), as well as intact right-hemisphere prefrontal cortex (for placement of cathode), to rule out stimulation of lesioned areas, as confirmed by magnetic resonance imaging (MRI) or computer tomography (CT) scans.

*Exclusion criteria* are: Contraindications for tDCS (e.g., cardiac pacemaker, history of seizures, implanted metal inside the head); more than one clinically apparent stroke with aphasic symptoms, as documented by clinical records and MRI or CT scans of the brain; other severe neurological diseases, as assessed by an independent neurologist (e.g., epilepsy, brain tumor, subdural hematoma); psychiatric conditions that may impair effective communication, as evaluated by an independent clinical psychologist or psychiatrist according to the DSM-5 system: current diagnosis of severe alcohol or substance use disorder (at least six out of eleven symptoms), current major depressive episode (at least five out of nine symptoms, among them depressed mood or anhedonia), and current disorder from the psychotic spectrum (at least two out of five symptoms, among them delusions, hallucinations or disorganized speech) (14); severe apraxia of speech, as revealed by Hierarchical Word Lists (<20% phonetically and phonematically correct items) (15); severe non-verbal cognitive deficits, as demonstrated by the Corsi Block-Tapping Task (less than four correct items) (16); severely impaired vision or hearing that prevents individuals from engaging in intensive SLT based on clinical experience; and changes in centrally active drugs within 2 weeks prior to study inclusion.

### Randomization

**Figure 1:**
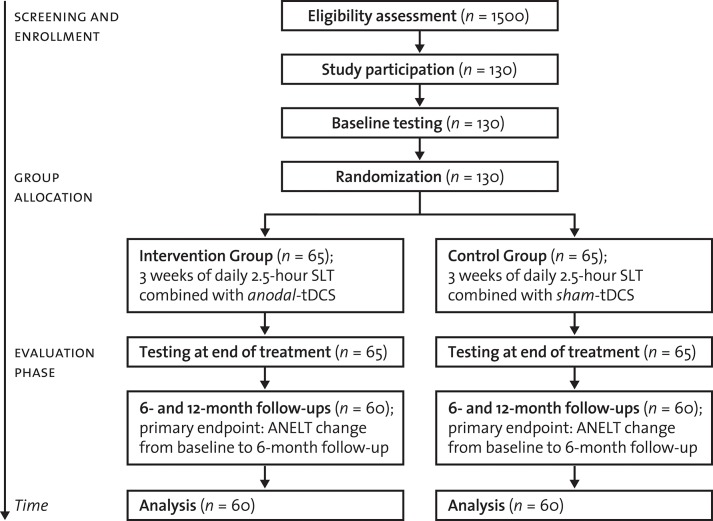
Trial flow. SLT, Speech-language therapy; tDCS, transcranial direct current stimulation; ANELT, Amsterdam-Nijmegen Everyday Language Test (A-scale).

In addition to intensive SLT, participants will either receive anodal-tDCS or sham-tDCS, depending on group assignment (allocation ratio of 1:1). Groups will be stratified-randomized according to age (<60 vs. ≥ 60 years), aphasia severity at baseline (AAT *t*-score classification at screening: severe vs. mild-to-moderate aphasic symptoms), and center (for details, see Figure 1). Block randomization will be used (randomly varying block sizes of 4 and 6).

### Behavioral Treatment, Stimulation, and Blinding

In the planned RCT, participants will receive intensive treatment (5) consisting of computer-assisted naming therapy (12) and face-to-face communicative-pragmatic therapy (17). Treatment will be tailored to the participants' individual needs. For naming therapy, personally relevant items will be chosen in a baseline screening to reflect individual language ability, a procedure previously described and established in a phase-II RCT (12). For communicative-pragmatic therapy, training tasks of individual complexity will be identified in an extensive baseline screening (18). These tasks will include a variety of situations known from everyday life that require verbal and non-verbal skills in context of social interaction (17). Treatment will be administered in two daily sessions over a period of three consecutive weeks (2 h of daily naming therapy; 30 min of daily communicative-pragmatic therapy; total weekly dosage: 12.5 h). During the entire 3-week treatment period, participants will not attend any other form of SLT.

Intensive SLT will be combined with tDCS using a battery-driven device (DC-Stimulator Plus, NeuroConn, Ilmenau, Germany). The anode (5 × 7 cm^2^) will be placed horizontally over the left M1 representation of the hand (C3 of the 10–20 international EEG system) (12, 19). A functionally inert cathode (10 × 10 cm^2^) will be positioned over the right supraorbital region. In the *Intervention Group*, participants will receive 20 min of excitatory anodal-tDCS of M1 administered at the beginning of each session. In the *Control Group*, the current will be ramped up and remain at 1 mA only for 30 s prior to ramping down. This sham-tDCS does not affect neural activity, but ensures blinding of participants due to initial tingling sensations on the scalp, and therefore is the comparator of choice consistent with previous work (12, 19). Participants and therapists will be blinded to group assignment by using the “study mode” of the stimulator, a masked randomization procedure determined by algorithms. The randomization sequence will be encrypted and stored on a secure server. Crucially, participants and therapists will be unaware of the group assignment throughout the entire study. To further increase blinding integrity, data evaluation will be performed by an independent Endpoint Committee blinded to group assignment and time of assessment.

### Hypotheses

The *primary hypothesis* predicts that intensive SLT combined with anodal-tDCS leads to better communication ability than intensive SLT combined with sham-tDCS. Likewise, *secondary hypotheses* predict favorable scores on secondary outcomes with anodal-tDCS compared to sham-tDCS (for details, see Table 1). *Primary endpoint* will be a 6-month follow-up; *secondary endpoints* will be immediately after treatment, and a 12-month follow-up. The 6-month follow-up as primary endpoint is assumed to reflect the impact of symptom recovery on aspects of everyday life most adequately and proved to be particularly sensitive to the effect of anodal-tDCS in previous work (12). The 12-month follow-up will help explore the long-term stability of the expected results.

**Table 1:** Testing.

**Study procedure**	**Pre-screening**	**Screening**	**Baseline**	**Treatment**	**Post-treatment**	**6-month follow-up**	**12-month follow-up**
**Visit number**			**T**_**0**_		**T**_**1**_	**T**_**2**_	**T**_**3**_
**Place**	**Clinic**	**Clinic/patients' home**	**Clinic/patients' home**	**Clinic**	**Clinic/patients' home**	**Clinic/patients' home**	**Clinic/patients' home**
Study month			0	Week 1–3	1	6	12
Informed consent	•						
In- and exclusion criteria (chart-based)	•						
In- and exclusion criteria (study-specific)		•					
Demographics and medical history		•					
Prior and current treatment		•					
Vital signs				•			
Physical examination				•			
Magnetic resonance imaging		•					
Aachen Aphasia Test		•				•	
Hierarchical Word Lists		•					
Corsi Block-Tapping Task		•					
Amsterdam Nijmegen Everyday Language Test			•		•	•	•
Naming ability		•			•	•	•
Communicative- Pragmatic Screening			•				
Scenario Test			•			•	
Communicative Effectiveness Index			•		•	•	•
NeuroCogFX			•			•	
Benton Visual Retention Test			•			•	
SADQH-10			•		•	•	•
SAQOL-39g			•		•	•	•
EQ-5D-5L and EQ-5D-VAS			•		•	•	•
Patient Resource Consumption Questionnaire			•			•	•
Burden of informal caregivers			•		•	•	•
tDCS Safety Questionnaire			•	•	•	•	•
Minimal Important Difference					•	•	•

*Selected linguistic functions, Aachen Aphasia Test (13); Apraxia of speech, Hierarchical Word Lists (14); Non-verbal working memory, Corsi Block-Tapping Task (15); Communication ability (primary outcome), Amsterdam Nijmegen Everyday Language Test (A-scale; parallel versions used in counterbalanced order across participants) (6); Naming ability, personally relevant trained and untrained items, consistent with previous work (12); (Non-)verbal communication, Communicative-Pragmatic Screening (18), Scenario Test (Nobis-Bosch et al. in preparation), and Communicative Effectiveness Index (20); Attention and executive function, reaction-speed subscales from NeuroCogFX (21); Non-verbal episodic memory, Figure Recognition Task from Benton Visual Retention Test (22); Mood, German version of the 10-item Stroke Aphasic Depression Questionnaire (SADQH-10) (23); Health-related quality of life, SAQOL-39g (24); EQ-5D-5L and EQ-5D-VAS (25); Health economic evaluation: direct and indirect costs during the 12-month study period (determined by the self-developed Patient Resource Consumption Questionnaire and based on common standardized unit cost assumptions), and Quality-Adjusted Life Years (derived from the EQ-5D-5L data) (26); Unpaid support provided by family members or friends, Burden of informal caregivers (27); (Serious) adverse events, tDCS Safety Questionnaire (28); Subjective treatment-induced change, as experienced by patients, therapists, and relatives, Minimal Important Difference (29)*.

### Primary Outcome

Communication ability in 10 everyday-life situations will be evaluated by parallel versions of the ANELT (A-scale) (6). ANELT has good psychometric properties, including high test-retest reliability and sensitivity to treatment-induced progress in chronic post-stroke aphasia (2, 5). Each testing session will be videotaped and scored offline by the external Endpoint Committee.

### Secondary Outcomes

*Secondary* endpoints include measures representing linguistic-executive skills, attention, memory, emotional well-being, quality of life, health economic costs, and adverse events (for details, see Table 1).

### Sample Size Estimates

We expect a between-group difference on the ANELT of at least three points at the 6-month follow-up. An independent-sample *t*-test with a two-sided significance level of 0.05 has a power of 80% to detect an average between-group difference of 3.0 points (standard deviation: 5.8) at the 6-month follow-up if data from 120 participants are analyzed, according to calculations using nQuery with raw data from previous work (12). Assuming a drop-out rate of 10%, 130 individuals will be recruited. Determining samples sizes with *t*-tests is a conservative approach: the planned analysis of covariance (ANCOVA) with (1–*p*^2^) × *n* participants has the same power as a *t*-test with *n* participants, where *p* is the variance deflation factor, calculated by the correlation of baseline and follow-up measures (30). In the worst case of *p* = 0, the resulting sample size corresponds to a *t*-test.

### Statistical Analyses

Focusing on the *primary* hypothesis, we will conduct an ANCOVA with the 6-month follow-up ANELT scores as dependent variable, with group assignment (anodal-tDCS or sham-tDCS) as independent variable, and baseline ANELT scores as covariate. Further covariates are the stratification parameters specified above (i.e., age, aphasia severity at baseline, and center). The trial outcome will be analyzed on an “intention-to-treat” basis in the total group. In the event of missing values, multiple imputation methods will be applied, if appropriate. Regarding the *secondary* hypotheses, data will be analyzed using standard statistical methods while adjusting for possible confounders, including the stratification parameters. As participants may differentially benefit from anodal-tDCS of M1 depending on type of aphasia, exploratory subgroup analyses will determine response rates related to individual syndromes.

### Safety, Protocol, and Data Monitoring

Safety, protocol, and data monitoring will be conducted by the Clinical Trials Coordination Center of the University Medicine Greifswald. For constant tDCS applied over cortical areas in awake humans, potential minor side effects are skin irritation, initial phosphenes, headache, dizziness, and itching under the electrode. Intensity, duration and frequency of tDCS are within the limits suggested in current guidelines (31). Number and type of adverse events will be diagnosed by a study physician, reported to the Clinical Trial Management within seven days, and classified using a standard questionnaire (28). In addition, serious adverse events will be reported to an independent Data Safety and Monitoring Board that will advise whether to continue, modify or stop the treatment and decide whether to unmask the group assignment. To ensure treatment fidelity and participant compliance, all training sessions will be audiotaped (naming therapy) or documented in written form (communicative-pragmatic therapy). These materials will be continuously evaluated by the Clinical Trial Management. Moreover, the Data Safety and Monitoring Board will participate in annual meetings with the Clinical Trial Management to ensure adherence to the study protocol, including blinding integrity. All primary and secondary outcomes will be scored by the external Endpoint Committee blinded to group assignment and time of assessment. Access to the dataset and stimulator-generated randomization sequence will be restricted to the Clinical Trial Management and the Data Safety and Monitoring Board. To protect individual privacy before, during and after the trial, the dataset will be stored, analyzed and archived in a pseudonymized manner.

## Discussion

The proposed protocol of a phase-III RCT aims to demonstrate that intensive SLT combined with anodal-tDCS benefits verbal communication and parameters related to activities of daily living in chronic post-stroke aphasia. Previous phase-II RCT data have shown that naming therapy combined with anodal-tDCS improves performance of trained items and, to a smaller degree, untrained items alongside proxy-rated communication ability (12, 19). Progress in communication ability may have a positive impact on associated factors, such as severity of post-stroke depression. Thus, the current RCT considers the overall outcome of intensive SLT on measures evaluating linguistic-executive skills, attention, memory, emotional well-being, quality of life, and health economic costs. In- and exclusion criteria are kept liberal to allow generalization of the expected results to realistic routine-healthcare conditions. The age split of 60 years for randomization was chosen to control for age-dependent cerebral changes, such as microangiopathy or reduced neuroplasticity. Covering both utterance-centered and communicative-pragmatic treatment strategies, the selected SLT methods reflect best-practice guidelines in aphasia rehabilitation (5).

There are two major reasons for stimulating M1 instead of individually determined intact perilesional brain regions. *First*, perilesional stimulation requires pre-treatment mapping of preserved language-related neural activity. Such an approach is time-consuming, expensive, and involves technical expertise usually not available outside specialized research centers. *Second*, the rationale for anodal-tDCS of M1 comes from neuroscience evidence suggesting that the motor system is anatomically and functionally linked with perisylvian eloquent areas (32–34). Moreover, behavioral studies revealed that engaging the primary motor cortex facilitates language processing in healthy individuals (35–37) and in persons with aphasia (38–43). Accordingly, phase-II RCT data confirm that anodal-tDCS of M1 leads to elevated naming and communication performance in chronic post-stroke aphasia (12) while increasing activity and connectivity in the preserved language network (19). Although the neural bases of recovery from aphasia are not fully understood, anodal-tDCS of M1 may affect sensorimotor coupling via pathways from posterior language regions (44) or alter connectivity of motor and prefrontal areas relevant to speech (45). Current spread may also change excitability in premotor or prefrontal regions (46) and hence support domain-general cognitive function, resulting in better language performance across different aphasia subtypes, severity levels, and lesion sites (47). To confirm the local influence of anodal-tDCS on M1 and to investigate the underlying neural mechanisms, an add-on study will focus on individualized modeling of current flow in a subset of rehabilitation centers with access to high-resolution MRI.

In conclusion, intensive SLT can relieve symptoms in chronic post-stroke aphasia, but effect sizes are moderate (5). This highlights the need to explore adjunct strategies, such as anodal-tDCS, to enhance training effectiveness. Recently, we provided phase-II RCT data indicating that intensive SLT combined with anodal-tDCS of M1 benefits naming and communication ability in chronic post-stroke aphasia, with medium-to-large effect sizes (12). The present study seeks to establish anodal-tDCS of M1 by substantiating our previous findings in a phase-III RCT with adequate power, ecologically valid outcomes, and evidence-based SLT. After trial completion, a workshop with relevant stakeholders will ensure transfer into best-practice guidelines and successful integration within clinical routine.

## Ethics Statement

The present study protocol was approved by the Ethics Review Board of the University Medicine Greifswald, Germany (reference number: BB-013/18). All participants will provide written informed consent.

## Author Contributions

BS, RD, VP, MM, UG, TR, TG, CB, and AF: study concept, design, and manuscript revisions. BS, RD, MM, UG, TG, and AF: funding acquisition. BS, VP, and AF: manuscript drafting. BS: artwork. All authors read and approved the final manuscript.

### Conflict of Interest

The authors declare that the research was conducted in the absence of any commercial or financial relationships that could be construed as a potential conflict of interest.

## Abbreviations

AAT: Aachen Aphasia Test
ANCOVA: analysis of covariance
ANELT: Amsterdam-Nijmegen Everyday Language Test
M1: primary motor cortex
RCT: randomized controlled trial
SLT: speech-language therapy
tDCS: transcranial direct current stimulation.
